# Age-Related Alterations in Swallowing in Healthy Aged Rodents: A Systematic Review

**DOI:** 10.3390/biology15110835

**Published:** 2026-05-26

**Authors:** Han-Na Kim, Ji-Youn Kim

**Affiliations:** 1 Department of Dental Hygiene, Yonsei University, Wonju-si 26493, Republic of Korea; hannakim@yonsei.ac.kr; 2 Department of Dental Hygiene, Gachon University, Incheon 21936, Republic of Korea

**Keywords:** deglutition disorders, healthy aging, rodentia, swallows

## Abstract

Age-related decline in swallowing function is increasingly recognized as a clinical concern in aging populations. To better understand the biological mechanisms underlying functional and structural changes associated with normal aging, rodent models provide a controlled experimental platform. This review systematically examined age-related changes in swallowing function in healthy rodents, compared with that of young adults. A comprehensive literature search was conducted in PubMed, Embase, and the Cochrane Library. Twenty-nine articles were ultimately included in this review. Age-related alterations in swallowing function in healthy aged rodents were reported in the following categories: contraction properties of tongue muscles, histomorphological and biochemical changes in tongue muscles, swallowing-related cranial sensorimotor system, histological changes in pharyngeal muscles, and swallowing behavioral outcomes. The evidence summarized in this review provides insights into the functional, histological, and behavioral alterations in swallowing in healthy aged rodents compared with those of young adult rodents. These findings enhance the understanding of the biological basis of presbyphagia and support the translational value of rodent models in developing preventive and rehabilitative strategies for age-related dysphagia.

## Simple Summary

Swallowing problems become more common with aging, even in otherwise healthy older adults, and can impair nutritional status, health, and quality of life. Rodent models provide an important experimental platform for investigating the biological mechanisms underlying these changes. This systematic review summarizes evidence from healthy aged rodent studies examining age-related alterations in swallowing-related structures and functions. The reviewed studies reported changes in tongue muscle properties, pharyngeal muscle histology, swallowing-related cranial sensorimotor systems, and swallowing behaviors, suggesting that aging affects multiple components of the swallowing mechanism.

## 1. Introduction

Dysphagia is a common geriatric syndrome, affecting many older adults worldwide with impaired swallowing function [1]. It is frequently observed in older adults with neurodegenerative diseases such as Parkinson’s disease and Alzheimer’s disease, as well as in those with stroke [1]. However, even healthy older adults without any specific diseases may experience structural, physiological, or neural changes in the swallowing mechanism [1]. These changes can affect the pressure and speed of swallowing movements [1]. Presbyphagia refers to an age-related decline in the swallowing function that can occur as part of the natural aging process, even in the absence of specific diseases [2,3,4,5]. This condition affects approximately 40% of healthy individuals over the age of 60 [6]. Aging is often perceived as normal; however, awareness and interest regarding the impact of aging on swallowing function are generally lacking. If presbyphagia is left untreated, it can result in reduced food intake, deterioration of overall health, severe complications, and negative effects on mental well-being, thereby ultimately diminishing a person’s quality of life [1]. Therefore, to improve and preserve the swallowing function in healthy older adults, it is essential to accurately identify the symptoms of geriatric dysphagia in the aging population and the age-related alterations in swallowing biomechanics. A precise understanding of aging-related changes in swallowing mechanisms will enable the development of better treatment strategies for older adults suffering from dysphagia.

The swallowing process involves multiple organ systems, including the nervous and musculoskeletal systems. Normal swallowing requires the coordinated effort of more than 30 muscles and the nervous system. Additionally, the key anatomical structures involved in swallowing include the oral cavity, pharynx, and esophagus. Therefore, neurological, muscular, and physiological age-related changes are known to affect swallowing function in older adults [1]. With aging, neurodegeneration can occur owing to changes in neural structures, neurochemicals, and related functions. Studies in human subjects have reported that healthy older adults show increased activation across a broader range of cortical regions during swallowing [7]. In addition, reduced activation in somatosensory cortical regions in older adults has been interpreted as an age-related decline in sensory processing and sensorimotor integration, potentially leading to the compensatory recruitment of additional cortical areas [8]. Moreover, the total muscle mass in older adults decreases by approximately 0.5–1% per year, leading to a 30–50% reduction by the age of 80 [9]. Since numerous muscles are involved in the coordinated physiological action of swallowing [10], age-related muscle changes such as decreased muscle mass and contraction strength may contribute to swallowing difficulties. As mentioned previously, all physiological functions of the body gradually decline with age, which may be a potential cause of dysphagia [11]. This functional decline is attributed to the characteristic molecular and cellular changes associated with normal aging [12]. However, studying these neurological and muscular changes at the molecular and cellular levels in human subjects poses significant challenges. Functional assessments of age-related swallowing impairment in humans are difficult to conduct. To overcome these limitations, various scientific studies have used animal models as substitutes for human research. Given these challenges, animal models provide a valuable alternative for investigating the underlying mechanisms of age-related changes in swallowing. The aim of this review was therefore to systematically summarize the findings of studies that have investigated age-related changes in swallowing biomechanics by using rodent models.

## 2. Materials and Methods

### 2.1. Searching Strategy

**Figure 1:**
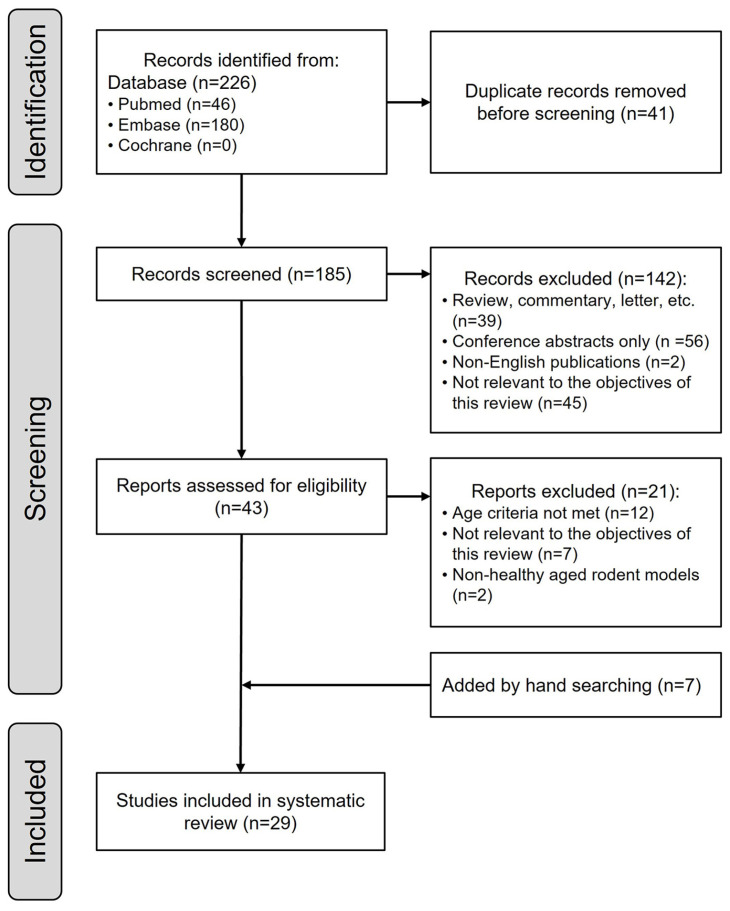
Flowchart of the systematic literature search and selection process. The diagram illustrates the identification, screening, eligibility assessment, and inclusion of studies according to the Preferred Reporting Items for Systematic Reviews and Meta-Analyses (PRISMA) guidelines.

This systematic review was registered with PROSPERO (CRD420261303942). The study adhered to the Preferred Reporting Items for Systematic reviews and Meta-Analyses (PRISMA) guidelines. The search criteria were established to identify articles that evaluated age-related changes in swallowing function in healthy aging rodent models. The search strategy combined keywords related to rodents, dysphagia, and aging, including (“rodent” OR “mouse” OR “rat”) AND (“dysphagia” OR “swallowing disorder” OR “deglutition disorder”) AND (“aging” OR “age-related” OR “senescence”). A comprehensive database search was conducted in January 2026 using PubMed, Embase, and the Cochrane Library. The search was limited to the title and abstract fields. Reference lists of the included articles were also manually screened to identify additional relevant articles. A total of 226 articles were retrieved: 46 from PubMed, 180 from Embase, and none from the Cochrane Library. After removing 41 duplicate articles, a final total of 185 articles remained (Figure 1).

### 2.2. Inclusion and Exclusion Criteria

Screening and eligibility assessments were conducted independently by two reviewers (J-Y Kim and H-N Kim) using predefined inclusion and exclusion criteria. The criteria used to select relevant studies are summarized in Table 1. A total of 45 studies were excluded because they were not relevant to the focus of the review, 56 studies were excluded as conference abstracts, 2 studies were non-English publications, and 39 studies were not original research articles. Consequently, of the 185 articles initially identified, 142 were excluded after title and abstract screening. The remaining 43 articles underwent a full-text assessment for eligibility (Figure 1). Based on previous studies, the age criteria for classifying rodents as aged were defined as rats older than 29 months and mice older than 18 months [13,14,15]. Therefore, only studies in which the rats were older than 29 months and the mice were older than 18 months at the end of the experimental period were included in this review. In studies in which three age groups (i.e., young adult, middle-aged, and old) were used, only the studies in which the oldest group met the defined age criteria were considered eligible, and only data from the young adult and old age groups were included in the current review. Studies evaluating the effects of interventions (e.g., tongue exercises and pharmacological treatments) on swallowing function in aged rodents were excluded. However, intervention studies were included if they provided data on nonintervention control groups that met the inclusion criteria. Furthermore, studies involving rodent models of disease (e.g., amyotrophic lateral sclerosis and Parkinson’s disease) were excluded. A total of 21 studies were excluded during the full-text eligibility assessment. Consequently, 29 studies were ultimately included in the final review, including 7 additional studies identified during the updated screening process (Figure 1).

### 2.3. Quality Assessment and Risk of Bias

**Figure 2:**
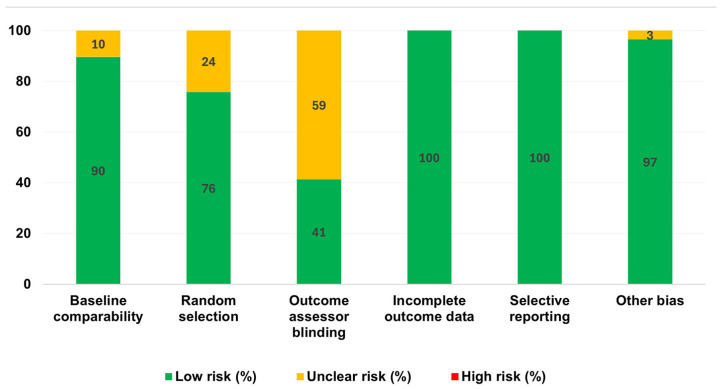
Risk of bias summary assessed using selected domains from the SYRCLE risk of bias tool. Bars indicate the proportion of studies judged as low, unclear, or high risk of bias across the evaluated methodological domains.

The methodological quality and risk of bias of the included studies were evaluated using selected domains from the risk of bias tool developed by the Systematic Review Centre for Laboratory Animal Experimentation (SYRCLE; Utrecht, the Netherlands), which was specifically designed to assess bias in animal intervention studies [16]. The original SYRCLE tool comprises 10 domains categorized into selection, performance, detection, attrition, reporting, and other sources of bias [16]. In the present review, six domains that could be clearly determined based on the methodological information reported in the included studies were evaluated: baseline comparability (selection bias), random selection for outcome assessment and outcome assessor blinding (detection bias), completeness of outcome data (attrition bias), selective outcome reporting (reporting bias), and other potential sources of bias. Domains related to performance bias were not evaluated, as the included studies did not involve experimental interventions. Therefore, these domains were not applicable to the study designs included in this review. Each domain was judged as having a low, high, or unclear risk of bias for each study. The results of the risk of bias assessment are presented in Figure 2. Two reviewers independently performed the assessment, and any disagreements were resolved through discussion.

## 3. Results

### 3.1. General Characteristics

**Figure 3:**
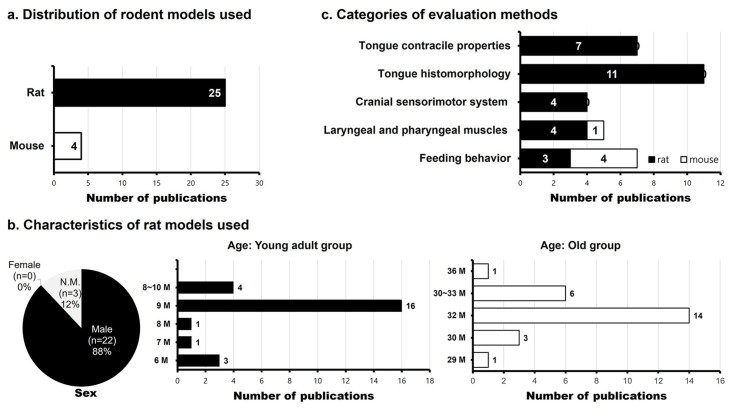
Overview of the included studies (**a**) Distribution of rodent models. (**b**) Sex and age distribution of rat models. (**c**) Categories of evaluation methods. M: months; N.M.: not mentioned.

Among the 29 selected studies, 25 studies used rats and 4 studies used mice (Figure 3). All 25 studies used Fischer 344/Brown Norway rats [17,18,19,20,21,22,23,24,25,26,27,28,29,30,31,32,33,34,35,36,37,38,39,40,41]. Of these, 22 studies used male rats, whereas three studies did not specify the sex of the animals [24,35,41]. In the 25 rat studies, the young adult group consisted of rats aged 6–10 months, with most studies using rats aged 9 months. The “old” group consisted of rats aged 29–36 months, and most studies used rats aged 32 months. Several studies used rats aged 6 months (i.e., “young adult”), 18 months (i.e., “middle-aged”), and 30 months (i.e., “old”). However, only the results of the young adult (6 months) and old (30 months) groups were considered in this review, in accordance with the objectives of the review. Among the four mouse studies [42,43,44,45], three studies used C57BL/6 mice and one study used FVB mice. The sex distribution varied: two studies included both male and female mice, whereas two studies used only males. In the rat studies, the age was consistent, whereas the age of the mice varied. In the young adult group, one study used mice younger than 2 months [44], whereas three studies used mice aged 4–7 months [42,43,45]. In the old group, the age ranges were 24 months [42], 18–21 months [43], 23–25 months [44], and 19 and 26 months [45], respectively. The 29 studies were categorized into five groups based on the targeted muscles or assessment method: (1) age-related changes in tongue contraction properties (7 rat studies), (2) age-related morphological and biochemical changes in the tongue muscles (11 rat studies), (3) age-related changes in the cranial sensorimotor system related to swallowing (4 rat studies), (4) age-related changes in the pharyngeal muscles (4 rat studies, 1 mouse study), and (5) age-related feeding behavior changes (3 rat studies, 4 mouse studies). If a study was included in multiple categories, it was listed in all relevant categories (Figure 3).

### 3.2. The Age-Related Changes in Tongue Muscle Contractile Property in Aged Rats

Seven studies that examined aging-related changes in tongue contraction properties used male Fischer 344/Brown Norway rats, with the “young adult” and “old” groups typically aged 9 months and 32 months, respectively. Studies reporting tongue contraction properties have focused on the extrinsic tongue muscles. Seven studies that investigated the aging-related changes in tongue contraction properties in rats were categorized based on the contraction actions of tongue retrusion and protrusion. To induce retrusion and protrusion tongue contraction actions, the hypoglossal nerve was stimulated in anesthetized young adult and old rats, and the tongue contractile properties of the extrinsic tongue muscles were recorded. Retrusive action in both age groups was reported by stimulating the whole hypoglossal nerve [17,18,19,20,21] or the lateral branch of the hypoglossal nerve [19]. Additionally, several studies reported the properties of protrusive action in both age groups induced by stimulation of the medial branch of the hypoglossal nerve [20,21,22,23]. Changes in the tongue contraction properties related to aging were confirmed by measuring five parameters: contraction time, half-decay time, maximal twitch force, maximal tetanic force, and fatigue index (Table 2).

### 3.3. The Age-Related Histomorphological and Biochemical Changes in Tongue Muscles of Aged Rats

The 11 studies that examined age-related histomorphological changes in the tongue were categorized based on whether they focused on extrinsic or intrinsic tongue muscles. Nine studies evaluated extrinsic tongue muscles, one study evaluated intrinsic tongue muscles, and one study evaluated both extrinsic and intrinsic tongue muscles. Table 3 summarizes the studies, based on the target muscles and in order of similar findings. All studies used Fischer 344/Brown Norway rats. With the exception of one study [24], all studies used male rats. The young adult and old groups were typically 9 months old and 32 months old, respectively. One study reported results only for the older group [27], and referenced previous research to characterize the young adult group as the control group [46]. The extrinsic tongue muscles evaluated were the genioglossus (GG), hyoglossus (HG), and styloglossus (SG). Most studies have targeted the GG muscle. Moreover, studies that examined aging-related changes in the myosin heavy chain (MHC) proportion in extrinsic tongue muscles were the most frequent, followed by studies that examined the muscle fiber cross-sectional area (CSA) and neuromuscular junction (NMJ) morphology. Among the extrinsic tongue muscles, NMJ evaluation was conducted only for the GG muscle. One study assessed histomorphological changes in intrinsic tongue muscles by examining changes in fiber size and MHC composition [29]. Another study identified the expression of satellite cells (SCs; i.e., adult muscle stem cells) in extrinsic and intrinsic tongue muscles in the young and old groups [30].

### 3.4. The Age-Related Changes in the Cranial Sensorimotor System in Aged Rats

Four studies investigated age-related changes in the cranial sensorimotor system of aged rats. Three studies focused on the hypoglossal nucleus, a cluster of motor neurons controlling tongue muscles, and one study examined the cortical motor area by using cortical microstimulation (Table 4). All studies used male Fischer 344/Brown Norway rats. The young adult group consisted of 9- to 10-month-old rats and the old group consisted of 32- to 33-month-old rats. Age-related morphological and neurochemical alterations in hypoglossal motoneurons, such as motoneuron size, number, and dendritic branching, have been observed [31], along with changes in serotonergic (5HT) input [32] and neurotrophic factors [33] in the hypoglossal nucleus. In addition, one study evaluated age-related changes in cortical plasticity [34].

### 3.5. The Age-Related Changes in the Laryngeal and Pharyngeal Muscles in Murine Models

Five studies were selected that examined age-related changes in the laryngeal muscles in rat and mouse models (Table 5). Four studies used Fischer 344/Brown Norway rats and all studies used male rats, except one study in which sex was not mentioned. One study used both male and female FVB mice. Among the four studies that used rats to examine age-related changes in laryngeal muscles, most studies focused on changes in the thyroarytenoid (TA) muscle among the four intrinsic laryngeal muscles (TA, posterior cricoarytenoid [PCA], lateral cricoarytenoid [LCA], and cricothyroid [CT]). One study showed changes in the MHC composition in all four intrinsic laryngeal muscles with aging [35]. Another study examined age-related structural and functional changes in the neuromuscular junctions (NMJs) in the TA and PCA muscles [36], while the other two studies assessed contractile dysfunction, metabolic profile [37], and MHC composition [38] in the TA muscle. The study using a mouse model evaluated muscle fiber size in three regions of the pharynx: the palatopharyngeus (i.e., naso- and oropharyngeal regions), thyropharyngeus, and cricopharyngeus (laryngopharyngeal region) [42].

### 3.6. The Age-Related Feeding Behavior Changes in the Aged Murine Models

Seven studies examined age-related changes in the feeding behavior of rats and mice (Table 6). Among the three studies using Fischer 344/Brown Norway rats, two studies used male rats [39,40], but the other study did not specify sex [41]. Two studies used videofluoroscopic swallow studies (VFSS) to assess age-related changes in feeding patterns and reported a decrease in mastication rate as the animals aged [39,41]. One study using feeding behavior monitoring demonstrated age-related alterations in bite number, inter-bite interval, and overall feeding duration in aged rats [40]. One study using FVB mice as the animal model included both female and male mice and reported a significant decrease in the lick rate with age [42]. Of the three studies using C57BL/6 mice, one study used female and male mice [43], whereas two studies used only male mice [44,45]. VFSSs in C57BL/6 mice consistently demonstrated age-related reductions in lick rate, although findings regarding pharyngeal transit time were not entirely consistent [43,45]. A study monitoring feeding behavior found significant differences in meal time and feeding bout values in an aged mouse group [44].

## 4. Discussion

The aim of this review was to summarize the effects of normal aging on the swallowing mechanism by analyzing how key swallowing structures in healthy, disease-free, and elderly rodents undergo functional and histomorphological changes compared to those in young rodents. In total, 29 selected studies predominantly used Fischer 344/Brown Norway rats as the animal model. Rodent models, such as rats and mice, have become indispensable in biomedical research and have served as substitutes for humans in various preclinical studies. Among the commonly used aging models, Fischer 344 (F344), Brown Norway (BN), and their hybrid Fischer 344 × Brown Norway F1 (F344/BN) are particularly favored because of their long lifespans and low disease incidences [13,47]. Most studies used male rats, with 9-month-old rats representing the young adult group and 30- to 32-month-old rats representing the old group. A 12-month-old rat corresponds to an approximately 30-year-old human, whereas a 30-month-old rat corresponds to a 75-year-old human [14]. Mice are similarly considered biologically mature at 8–12 weeks of age, while aging is defined as a minimum of 18 months [15]. Therefore, this review included only studies that examined rats aged 29 months or older and mice aged 18 months or older at the time of the experiment’s completion.

Although rodent models are widely used to investigate the mechanisms underlying age-related dysphagia, important anatomical and biomechanical differences exist between rodents and humans in the oral cavity, pharynx, and larynx. Compared with humans, rats possess a longer and less curvilinear oral cavity, a shorter pharynx, and a more rostrally positioned laryngeal complex. In addition, the ratio of cartilaginous to membranous vocal fold differs between the species, which may contribute to reduced susceptibility to aspiration in rodents. Behavioral differences in swallowing have also been reported. Rats typically perform successive swallows while a portion of the bolus remains within the oral cavity, whereas human swallowing during videofluorographic evaluation generally involves discrete bolus presentation and single swallows [39]. These species-specific anatomical and functional differences should be considered when interpreting translational findings from rodent swallowing studies. Despite these differences, rodent models reproduce several key features of human age-related swallowing dysfunction, including age-related changes in tongue contractile properties, sensorimotor control, bolus transport, and laryngeal and pharyngeal muscle function. Furthermore, many of the neural control mechanisms involved in swallowing, including brainstem swallowing circuits and cranial nerve-mediated sensorimotor pathways, are highly conserved across mammalian species. Therefore, rodent models remain valuable for investigating the cellular, neuromuscular, and behavioral mechanisms underlying dysphagia and for evaluating potential therapeutic interventions prior to human clinical studies.

### 4.1. Age-Related Changes in Tongue Contractile Properties

The tongue plays a crucial role in the oral phase of swallowing and consists of intrinsic and extrinsic muscles. Intrinsic tongue muscles (i.e., superior longitudinal, inferior longitudinal, transverse, and vertical muscles) modify tongue shape, whereas extrinsic tongue muscles (i.e., palatoglossus, genioglossus [GG], hyoglossus [HG], and styloglossus [SG]) control tongue movement and facilitate bolus transport [19]. The hypoglossal nerve innervates all intrinsic and extrinsic tongue muscles, except the palatoglossus. Protrusion is mediated by GG activation via the medial hypoglossal branch, while retraction is controlled by the HG and SG via the lateral branch [48]. Numerous studies have investigated the age-related changes in these extrinsic tongue muscles and focused primarily on the GG, HG, and SG muscles. However, only seven studies have compared healthy aging rodents with young adult rodents without tongue strength enhancement interventions. This review categorized tongue contractile properties into retrusive and protrusive actions. Tongue contraction was assessed by hypoglossal nerve stimulation, which measures five key muscle contractile properties.

The retrusive action is induced by stimulating the whole hypoglossal nerve or its lateral branch. Whole hypoglossal nerve stimulation coactivates both protrusive and retrusive muscles, generating net retraction [19,49,50]. Selective lateral branch stimulation of the hypoglossal nerve enhances retraction force [19,49,50]. Among the five studies on whole hypoglossal nerve stimulation, four studies reported significantly prolonged contraction times in the old group [17,18,19,21], whereas one study demonstrated no difference [20]. Half-decay times were significantly longer in two studies [17,21] but were unchanged in three studies [18,19,20]. No significant differences in the twitch force have been reported [17,18,19,20,21]. The tetanic force and fatigue resistance were unchanged in four studies [17,18,19,20] but significantly reduced in one study [21]. Selective lateral branch stimulation yielded no age-related differences in retrusive force [19]. These findings suggest that, while the temporal properties (contraction time and decay time) change with aging, the overall contraction force (i.e., twitch and tetanic forces) remains unaffected. Protrusive action is induced by medial branch stimulation. Two studies reported a reduced protrusive twitch force in aged rodents [21,23], whereas two other studies reported no significant differences [20,22]. The contraction time was significantly longer in one study [21] but unchanged in three studies [20,22,23]. Protrusive tetanic force was significantly lower in aged rodents in two studies [22,23] but unchanged in two other studies [20,21]. None of the four studies reported significant differences in the half-decay time or fatigue resistance [20,21,22,23]. These findings indicated some variability in age-related changes in protrusive tongue function. Although the included studies used broadly similar approaches to assess tongue contractile properties through hypoglossal nerve stimulation and tongue force recordings, several methodological parameters differed across studies. Variations included whole hypoglossal versus selective medial or lateral branch stimulation, medial or lateral branch transection, stimulation frequency and pulse parameters, fatigue paradigms, and anesthetic regimens. In addition, tongue positioning, preload tension, and force vector alignment varied across studies and may have influenced measured tongue force because of the length–tension properties of tongue muscles. Some studies individualized tongue positioning or stimulation intensity to optimize force recordings, whereas others used fixed stimulation paradigms. These methodological differences may partially explain the variability in tongue function findings across studies.

### 4.2. Age-Related Histomorphological and Biochemical Changes in Tongue Muscles

Age-related histomorphological and biochemical changes in the extrinsic tongue muscles have been widely studied, particularly with respect to myosin heavy chain (MHC) composition. In the GG, Type IIx was the most prevalent in the young and aged groups, followed by Type IIa, Type IIb, and Type I. Aged rodents had a significantly lower proportion of Type IIb MHC but a higher proportion of Type IIx and Type I MHC than those of the young controls [20,23,27]. These compositional shifts suggest a transition from fast, forceful contraction (Type IIb) toward slower or more fatigue-resistant muscle phenotypes (Type I and IIx), potentially reflecting a compensatory adaptation to age-related neuromuscular decline. These shifts may also contribute to age-related slowing of tongue movement and feeding behaviors. Reduced contraction speed during bolus propulsion may underlie decreases in mastication and lick rates observed in aged rodents. In HG and SG, Type IIx was the dominant isoform, with Type I being the least abundant. Connor et al. reported increased Type I MHC in aged HG [20], while Kletzien et al. observed decreased Type IIb and increased Type IIa MHC in aged HG [18]. In the SG, no significant age-related differences have been found [20] and the Type IIa proportions were increased in aged rodents [18]. The MHC compositional changes related to aging in the GG muscle showed similar results, whereas the findings for the HG and SG muscles showed somewhat different outcomes. These muscle-specific differences may be influenced by the distinct functional roles and usage patterns of each muscle during swallowing. The GG muscle plays an important role in tongue protrusion and bolus propulsion, whereas the HG and SG muscles are more involved in tongue retrusion and positional stabilization. Therefore, age-related compositional changes in the GG may have greater effects on protrusive tongue movement and bolus transport, whereas changes in the HG and SG muscles may more strongly influence swallowing coordination and timing than forceful tongue propulsion. Neuromuscular junction (NMJ) morphology in the GG is also affected by aging, with aged rodents exhibiting dispersed receptor clusters in the motor endplate [24]. Such changes may reflect impaired synaptic organization, which could contribute to reduced neuromuscular transmission efficiency. However, motor endplate size and volume remained unchanged, although the relationship between motor endplate and nerve terminal volume was disrupted in aged animals [24,25]. These findings suggest that structural connectivity between nerve terminals and muscle fibers may be altered by aging, even in the absence of overt atrophy. Additionally, studies examining the CSA of the GG and SG have reported no significant age-related differences [21,26,28], indicating that the muscle fiber size itself may be preserved despite underlying molecular or neuromuscular alterations.

In intrinsic tongue muscles, aging is associated with decreased Type IIb MHC and increased Type IIx and IIa MHC levels. Changes in muscle fiber CSA have been observed in the transverse and verticalis muscles [29], which are critical for shaping the tongue during bolus manipulation and propulsion. These alterations may contribute to age-related declines in the fine motor control of the tongue. Although maximum isometric tongue pressure is likely more strongly influenced by extrinsic tongue muscles, these age-related changes in intrinsic tongue muscles may also contribute to reductions in maximum isometric tongue pressure by affecting tongue shaping and stiffness during pressure generation. One study reported the altered expression of myogenic markers in intrinsic and extrinsic tongue muscles [30], suggesting potential impairments in muscle regeneration capacity with aging.

### 4.3. Age-Related Changes in Cranial Sensorimotor System, Pharyngeal Muscles, and Feeding Behaviors

Several studies have examined age-related changes in the cranial sensorimotor system, including hypoglossal motor neuron morphology, serotonergic input, and neurotrophic factor expression. Another study investigated the cortical motor plasticity by using microstimulation. Specifically, morphological evaluation of hypoglossal motoneurons revealed a significant reduction in the number of primary dendrites with aging, although the overall number and size of motoneurons remained unchanged [31]. Serotonergic (5HT) immunoreactivity did not differ significantly between age groups across all rostrocaudal levels of the hypoglossal nucleus [32]. In contrast, neurotrophic signaling showed partial age-related alterations: while BDNF expression was unaffected, TrkB immunoreactivity significantly decreased in both caudal and rostral regions of the nucleus [33]. Furthermore, cortical mapping using intracortical microstimulation demonstrated no significant age-related differences in the organization or excitability thresholds of motor areas controlling tongue and jaw movements [34]. These findings suggest that while gross cortical motor organization remains stable with aging, selective neurochemical and dendritic changes may occur in brainstem nuclei involved in swallowing control.

Most studies evaluating the pharyngeal muscles have focused on the thyroarytenoid (TA) muscle among the intrinsic laryngeal muscles (i.e., PCA, TA, LCA, and CT). MHC composition, NMJ morphology, CSA, and contractile properties were evaluated. Among these, Nagai et al. reported age-related MHC shifts in intrinsic laryngeal muscles, with increased Type IIa and IIx and decreased Type IIb MHC in the TA and LCA muscles, and elevated Type I MHC in the CT muscle [35]. McMullen and Andrade observed reduced NMJ density and altered acetylcholine receptor subunit expression in the TA and PCA muscles of aged rats, indicating partial denervation [36]. Age-related fragmentation of acetylcholine receptor clusters and reduced NMJ density may impair neuromuscular transmission and contribute to progressive motor unit denervation in aging skeletal muscle. Persistent or incomplete reinnervation may subsequently lead to contractile dysfunction and impaired motor coordination [36]. Functional deficits in the aged TA muscle, including reduced contractile force, slower shortening velocity, decreased fatigue resistance, and increased mitochondrial accumulation, were also demonstrated [37]. Kletzien et al. found reduced Type IIL (superfast) MHC in the aged TA, suggesting a decline in rapid muscle activity [38]. The reduction in fast-contracting Type IIb and Type IIL MHC isoforms, together with a relative increase in slower MHC phenotypes, may indicate selective vulnerability of fast motor units to aging-related denervation and metabolic stress. Increased mitochondrial accumulation in aged TA muscles may further represent a compensatory response to impaired oxidative metabolism and reduced contractile efficiency associated with aging. In mice, Randolph et al. reported age-related decreases in myofiber size across pharyngeal regions [42]. These findings collectively suggest that aging impairs the structural and functional integrity of pharyngeal and laryngeal muscles involved in swallowing through neuromuscular remodeling, altered muscle fiber composition, impaired metabolic adaptation, and progressive decline in fast motor unit function.

Aging-related changes in feeding behavior are commonly assessed using VFSS. In rat models, aging was associated with reduced mastication rate and bolus transport speed, with inconsistent findings regarding bolus area [39,41]. In B6 mice, aging led to slower swallowing across oral, pharyngeal, and esophageal phases, accompanied by increased bolus area and reduced lick rate, although pharyngeal transit time did not significantly differ with age [43,45]. Similarly, FVB mice demonstrated age-related declines in lick rate [42]. Studies monitoring feeding behaviors further revealed alterations in bite patterns and meal structure, characterized by prolonged feeding duration, increased bout frequency, and reduced bout size [40,44]. Notably, aged rats also exhibited reduced tongue base retraction and compensatory head movements during swallowing, suggesting biomechanical adaptations to age-related motor decline [41]. Collectively, these findings indicate that aging affects multiple aspects of feeding behavior and swallowing coordination in rodent models.

Collectively, the findings of this review suggest that age-related histomorphological and biochemical alterations in the tongue, pharyngeal, and laryngeal muscles, together with changes in tongue contractile properties and cranial sensorimotor regulation, may contribute to the swallowing behavioral changes observed in aging rodents. In particular, age-related shifts from fast-contracting MHC fibers toward slower or more fatigue-resistant phenotypes may partly underlie reduced contraction speed and altered tongue movement during swallowing. These histomorphological changes are consistent with VFSS findings demonstrating a reduced mastication rate, decreased lick rate, slower bolus transport, prolonged feeding duration, and reduced tongue base retraction in aged rodents. In addition, age-related alterations in neuromuscular junction morphology and sensorimotor regulation may further impair the temporal coordination of swallowing movements. From an oral rehabilitation perspective, the observed age-related alterations in tongue contractile properties, muscle composition, and feeding behavior may have important clinical implications. Reduced mastication efficiency, prolonged feeding duration, and altered bolus transport may increase the risk of oral residue and compromise safe swallowing in older adults. Although gross muscle atrophy was not consistently observed, neuromuscular and biochemical changes suggest a decline in functional reserve. These findings support the rationale for early intervention strategies targeting tongue strength, sensorimotor coordination, and oral muscle endurance. Furthermore, standardized functional outcome measures in rodent models may facilitate translational research aimed at optimizing rehabilitative protocols for presbyphagia. To date, studies examining functional and histomorphological changes in key swallowing structures of healthy aging rats have mostly been limited to the tongue, and the available data remain insufficient to establish consistent quantitative trends. Therefore, further research is required to better elucidate the complexity of presbyphagia. Discrepancies across studies highlight the need for standardized methodologies and larger sample sizes to clarify age-related patterns. Continued investigation using well-characterized rodent models will be essential for advancing translational strategies and developing targeted interventions for age-related dysphagia. The findings of this review provide a valuable foundation for future research and therapeutic innovation in oral rehabilitation.

## 5. Conclusions

Age-related alterations in swallowing function observed in healthy rodent models provide important insights into the biological mechanisms underlying presbyphagia in older adults. The present review identified consistent functional and histomorphological changes in key swallowing structures, particularly in the tongue muscles, as well as age-related modifications in the pharyngeal muscles, cranial sensorimotor system, and feeding behavior. Although structural atrophy was not uniformly observed, neuromuscular and biochemical alterations suggest a decline in functional reserve that may compromise swallowing efficiency with aging. Collectively, these findings enhance our understanding of the pathophysiological basis of age-related swallowing decline and highlight the translational value of rodent models for investigating presbyphagia. Further well-designed and standardized studies are required to clarify inconsistent findings and to facilitate the development of evidence-based preventive and rehabilitative strategies in oral health care for the aging population.

## Figures and Tables

**Table 1:** Inclusion and exclusion criteria.

Inclusion Criteria	Exclusion Criteria
Peer-reviewed original research articles	Review articles, conference abstracts, editorials, etc.
Studies reporting altered swallowing function in healthy, disease-free aging rodents	Review articles, conference abstracts, editorials, etc.
Articles written in English	Non-English articles
Animal studies	Nonanimal studies
Rodents were classified as “aged”: for rats > 29 months old and for mice > 18 months	Rodents not meeting the age criteria
Studies that compared healthy aging rodents with young adult rodents	No comparative analysis with young adults
Studies included nonintervention control groups (in intervention studies)	Intervention-only studies without control data

**Table 2:** The age-related changes in tongue muscle contractile property in aged rats.

Author (Year)	Animal Model (Sex)	Age (Months)	Tongue Contraction (Stimulation Method)	Key Findings
Ota et al. (2005) [17]	Fischer 344/Brown Norway rats (male)	Young: 8–10 Old: 30–33	Retrusive (whole) ^a^	· The twitch contraction time and half-decay time were significantly longer in the old group than in the young group, but maximum tongue forces (twitch and tetanic tension) and fatigability did not differ significantly between the age groups.
Kletzien et al. (2018) [18]	Fischer 344/Brown Norway rats (male)	Young: 9 Old: 32	Retrusive (whole)	· Contraction time was significantly longer in the old group than in the young adult group.
Becker et al. (2015) [19]	Fischer 344/Brown Norway rats (male)	Young: 9 Old: 32	Retrusive (whole or lateral) ^b^	· In whole hypoglossal nerve stimulation, the old group exhibited significantly longer contraction time, whereas the decay time, twitch force, tetanic force, and fatigue ratio were not significantly different between the groups. · In selective stimulation of the lateral branch of the hypoglossal nerve, no significant differences existed in any tongue contractile properties between the age groups.
Connor et al. (2013) [20]	Fischer 344/Brown Norway rats (male)	Young: 9 Old: 32	Retrusive (whole) and Protrusive (medial) ^c^	· In whole hypoglossal nerve stimulation and in medial branch stimulation, no significant differences existed in tongue contractile properties between the age groups.
Russell and Connor (2014) [21]	Fischer 344/Brown Norway rats (male)	Young: 9 Old: 32	Retrusive (whole) and Protrusive (medial)	· In whole hypoglossal nerve stimulation, the old group had significantly lower maximum retrusive tetanic force, longer contraction time, longer half-decay time, and lower fatigue ratio (i.e., greater fatigue), compared with the young adult group; however, the twitch force remained unaffected by age. · In selective stimulation of the medial branch of the hypoglossal nerve, the old group exhibited lower protrusive tongue twitch forces and longer contraction times, but the maximum protrusive tetanic force, half-decay time, and fatigue ratio were not significantly different between the groups.
Nagai et al. (2008) [22]	Fischer 344/Brown Norway rats (male)	Young: 9 Old: 30–32	Protrusive (medial)	· Maximal tetanic forces for protrusive contractions of the tongue were significantly reduced in old rats. · Mean twitch force, contraction time, half-decay time, and a calculated measure of fatigability did not differ significantly between the age groups.
Kletzien et al. (2013) [23]	Fischer 344/Brown Norway rats (male)	Young: 9 Old: 32	Protrusive (medial)	· Tongue strength (i.e., maximal twitch and tetanic tension) was significantly reduced in the old group, compared with the young adult group. · No significant age-related differences existed in the contraction time or half-decay time.

^a^ Retrusive tongue action was elicited by bilateral electrical stimulation of the whole hypoglossal nerve. ^b^ Retrusive tongue action was elicited by bilateral stimulation of the whole hypoglossal nerve or by selective stimulation of the lateral branch of the hypoglossal nerve. ^c^ Protrusive tongue action was elicited by bilateral electrical stimulation of the medial branch of the hypoglossal nerve.

**Table 3:** The age-related histomorphological and biochemical changes in tongue muscles of aged rats.

Author (Year)	Animal Model (Sex)	Age (Months)	Target Muscle/Evaluation Methods		Key Findings
Hodges et al. (2004) [24]	Fischer 344/Brown Norway rats (-) ^a^	Young: 9 Old: 36	ET	GG/IHC (NMJ morphology)	· Normal axon terminals and receptor clusters were clearly identifiable in the GG muscle of the young adult group, whereas the old group exhibited significantly greater receptor dispersion.
Johnson et al. (2011) [25]	Fischer 344/Brown Norway rats (male)	Young: 9 Old: 32		GG/IHC (NMJ morphology)	· Motor endplate volume did not differ significantly between the young adult and old groups. · In old animals, the relationship between motor endplate and nerve terminal volume was disrupted, whereas in the young group, nerve terminal volume increased proportionally with motor endplate volume.
Connor et al. (2009) [26]	Fischer 344/Brown Norway rats (male)	Young: 9 Old: 32		GG/IHC-CSA	· GG muscle fiber CSA did not differ significantly between the young adult and old groups.
Russell and Connor (2014) [21]	Fischer 344/Brown Norway rats (male)	Young: 9 Old: 32		GG/Masson’s trichrome staining (CSA)	· No significant aging effect was observed in GG muscle CSA. · The old group exhibited a significantly larger area of fibrosis in the GG muscle.
Schaser et al. ^b^ (2011) [27]	Fischer 344/Brown Norway rats (male)	Young: 9 [46] Old: 32		GG/SDS-PAGE, WB (MHC)	· The old group exhibited a significantly smaller proportion of Type IIb MHC in the anterior, medial, and posterior regions of the GG muscle compared to the young adult group, while the proportion of Type IIx MHC was significantly greater. · In the medial region of the GG muscle, the old group had a significantly greater proportion of Type I MHC compared to the young adult group. · No significant differences were observed in Type IIa MHC between the young adult and old groups.
Kletzien et al. (2013) [23]	Fischer 344/Brown Norway rats (male)	Young: 9 Old: 32		GG/SDS-PAGE (MHC)	· The old group exhibited a significantly greater proportion of Type I and IIx MHC and a significantly smaller proportion of Type IIb MHC compared to the young adult group.
Connor et al. (2013) [20]	Fischer 344/Brown Norway rats (male)	Young: 9 Old: 32		GG, SG, HG/SDS-PAGE (MHC)	· Type IIx MHC accounted for the largest proportion of each muscle (GG, HG, and SG) across all age groups, whereas Type I MHC accounted for the smallest proportion. · In the GG muscle, the old group had a greater proportion of Type I MHC and a smaller proportion of Type IIb MHC compared to the young adult group. · In the HG muscle, the old group exhibited a greater proportion of Type I MHC compared to the young adult group. · In the SG muscle, no statistically significant differences were found between age groups.
Kletzien et al. (2018) [18]	Fischer 344/Brown Norway rats (male)	Young: 9 Old: 32		SG, HG/SDS-PAGE (MHC)	· In the HG muscle, the old group exhibited a greater proportion of Type IIa MHC and a smaller proportion of Type IIb MHC compared to the young adult group. · In the SG muscle, the proportion of Type IIa MHC was higher in the old group compared to the young adult group.
Kletzien et al. (2018) [28]	Fischer 344/Brown Norway rats (male)	Young: 9 Old: 32		GG, SG, HG/TUNEL, WB, IHC (cell apoptosis, CSA)	· Aging significantly increased the index of cell death in the GG, SG, and HG muscles. · Caspase-3 and Bcl-2 protein expression (apoptosis regulators) increased in the extrinsic tongue muscles (GG, SG, HG) of old rats compared to young adults. · With aging, muscle fiber number significantly decreased in the old group, while muscle fiber diameter and CSA remained unchanged in the GG and SG muscles. (HG was excluded from muscle fiber morphometric analyses.)
Cullins and Connor (2017) [29]	Fischer 344/Brown Norway rats (male)	Young: 9 Old: 32	IT	IT/IHC (MHC, CSA)	· In the old group, the proportion of Type IIb MHC decreased, while the proportions of Type IIx and IIa MHC increased. · Age-related reductions in Type IIb and IIx muscle fiber size were observed only in specific intrinsic tongue muscles (transverse and verticalis).
Kletzien et al. (2020) [30]	Fischer 344/Brown Norway rats (male)	Young: 7 Old: 30	ET and IT	GG, SG, HG, IT/RT-qPCR, WB- ICC (SCs markers, p16INK4a)	· Pax7 (SC quiescence/activation marker) gene and protein expression significantly decreased with age in the GG muscle but remained unchanged in the SG, HG, and IT muscles. · p16INK4a (senescence marker) gene expression increased in the old GG, while protein expression remained unchanged across all tongue muscles. In Pax7-positive SCs isolated from the GG, p16INK4a expression increased with age. · No age-related changes were observed in the gene or protein expression of SC markers (MyoD [activated SC marker], myogenin [SC differentiation marker]), or p16INK4a in any tongue muscles.

ET: extrinsic tongue muscle; IT: intrinsic tongue muscle; GG: genioglossus; HG: hyoglossus; SG: styloglossus; NMJ: neuromuscular junction; MHC: myosin heavy chain; CSA: cross-sectional area; IHC: immunohistochemistry; SDS-PAGE: sodium dodecyl sulfate polyacrylamide gel electrophoresis; WB: Western blotting; RT-qPCR: reverse transcription quantitative polymerase chain reaction; SC: satellite cell; MyoD: myoblast determination protein. ^a^ The minus symbol (-) indicates “not mentioned.” ^b^ Reference [27] used reference [46] as the control group.

**Table 4:** The age-related changes in the cranial sensorimotor system in aged rats.

Author (Year)	Animal Model (Sex)	Age (Months)	Evaluation Target/Method	Key Findings
Schwarz et al. (2009) [31]	Fischer 344/Brown Norway rats (male)	Young: 9–10 Old: 32–33	Hypoglossal nucleus/IHC (neuronal marker NeuN)	· The number of primary dendrites of hypoglossal motoneurons significantly decreased with age, while no age-associated changes were observed in the number or size of hypoglossal motoneurons.
Behan et al. (2012) [32]	Fischer 344/Brown Norway rats (male)	Young: 9–10 Old: 32–33	Hypoglossal nucleus/IHC (5HT)	· No significant age-related changes were found in 5HT immunoreactivity across the rostral, middle, and caudal regions of the hypoglossal nucleus.
Schaser et al. (2012) [33]	Fischer 344/Brown Norway rats (male)	Young: 9–10 Old: 32–33	Hypoglossal nucleus/IHC (BDNF, TrkB)	· BDNF levels in the ventral medial portion of the hypoglossal nucleus were not affected by age. · TrkB immunoreactivity significantly decreased with age in both the caudal and rostral regions of the hypoglossal nucleus.
Cullins et al. (2019) [34]	Fischer 344/Brown Norway rats (male)	Young: 9 Old: 32	Cortex motor area/intracortical microstimulation	· No significant age-related differences were observed in the cortical motor areas responsible for tongue and jaw movements. · No significant age-related differences were found in tongue or jaw motor thresholds.

IHC: immunohistochemistry; 5HT: 5-hydroxytryptamine (i.e., serotonin); BDNF: brain-derived neurotrophic factor; TrkB: tropomyosin receptor kinase B.

**Table 5:** The age-related changes in laryngeal and pharyngeal muscles in murine models.

Author (Year)	Animal Model (Sex)	Age (Months)	Evaluation Muscle/Method	Key Findings
Nagai et al. (2005) [35]	Fischer 344/Brown Norway rats (-) ^a^	Young: 8 Old: 30–32	PCA, TA, LCA, CT ^b^/SDS-PAGE (MHC)	· In the TA muscle of the old group, Type IIa/IIx MHC was increased, whereas Type IIb MHC was decreased, compared with that of the young adult group. · In the LCA muscle of the old group, Type IIb MHC was decreased compared with that of the young LCA muscles. · In the PCA muscle, no significant differences in MHC isoform proportions existed between the young adult and old groups. · In the CT muscle of the old group, Type I MHC was increased, compared with that of the young CT muscles.
McMullen and Andrade (2009) [36]	Fischer 344/Brown Norway F1 hybrid rats (male)	Young: 6 Old: 30	PCA and TA/IHC (NMJ), PCR and WB (AChR subunit ^c^), in vitro contractile function test	· In the TA muscles (a vocal fold adductor), NMJ density, size, quantity, and clusters were significantly decreased in the old group, compared with those of the young adult group. The mRNA expression of acetylcholine receptor (AChR) ε and γ subunits increased, whereas AChRδ mRNA expression was decreased in the old group. Protein expression of AChRγ and ε subunits were increased in the old group. · In the PCA muscles (the primary vocal fold abductor), NMJ density, size, quantity, and clusters were significantly decreased in the old group. The mRNA expression of the AChRα, γ, and δ subunits were increased in the old group. The protein expression of AChRε was increased, whereas AChRγ was decreased in the old group. · The PCA and TA muscles in the old group were weaker and more sensitive to tubocurarine (a neuromuscular blocker), which provided functional evidence of denervation.
McMullen and Andrade (2006) [37]	Fischer 344/Brown Norway F1 hybrid rats (male)	Young: 6 Old: 30	TA/modified Gomori’s trichrome and periodic acid Schiff staining and Isometric contractile properties recording	· TA muscle fiber size in the old group was approximately 59% greater than that of the young adult group. · Aging did not alter the time to peak twitch force but age increased the half-relaxation time by more than 30% in the old group. The twitch and peak tetanic forces were significantly lower in the old group, compared with the young adult group. · Maximal shortening velocity decreased by 20% in the old group, and the velocity of unloaded shortening was 27% slower than that of the young adult group. · Fatigue resistance was significantly decreased in the old group. The TA muscle in the old group contained fibers with abundant mitochondrial accumulations (i.e., ragged red fibers). Intracellular glycogen content in the TA muscles increased with age.
Kletzien et al. (2016) [38]	Fischer 344/Brown Norway rats (male)	Young: 9 Old: 32	TA/SDS-PAGE (MHC)	· With aging, Types I, IIx, and IIb MHC levels were increased, whereas Type IIL MHC (i.e., superfast) levels were significantly decreased in the TA muscle.
Randolph et al. (2014) [42]	FVB mice (male and female)	Young: 2 and 12 Old: 24	Pharyngeal region ^d^/H&E (CSA)	· From 2 to 12 months of age, myofiber size significantly increased in both the nasopharynx and oropharynx, while it decreased in the laryngopharynx. However, by 24 months of age, myofiber size significantly decreased in all three pharyngeal regions.

PCA: posterior cricoarytenoid; TA: thyroarytenoid; LCA: lateral cricoarytenoid; CT: cricothyroid; SDS-PAGE: sodium dodecyl sulfate polyacrylamide gel electrophoresis; MHC: myosin heavy chain; IHC: Immunohistochemistry; NMJ: neuromuscular junction; RT-qPCR: reverse transcription quantitative polymerase chain reaction; WB: Western blotting; H&E: hematoxylin and eosin; CSA: cross-sectional area. ^a^ The minus symbol (-) indicates “not mentioned.” ^b^ The intrinsic laryngeal muscles were the posterior cricoarytenoid (PCA), thyroarytenoid (TA), lateral cricoarytenoid (LCA), and cricothyroid (CT). ^c^ The AChR subunits were α, β, ε, γ, and δ. ^d^ This article divides the pharyngeal region into three regions: the palatopharyngeus (i.e., naso- and oropharyngeal regions), thyropharyngeus, and cricopharyngeus (i.e., laryngopharyngeal region).

**Table 6:** The age-related feeding behavior changes in the aged murine models.

Author (Year)	Animal Model (Sex)	Age (Months)	Evaluation Method	Key Findings
Russell et al. (2013) [39]	Fischer 344/Brown Norway rats (male)	Young: 9 Old: 32	VFSS/swallowing function	· The old group had a significantly slower mastication rate and bolus transport speed than that of the young adult group. · The old group had a significantly smaller bolus area than that of the young adult group.
Krekeler and Connor (2017) [40]	Fischer 344/Brown Norway rats (male)	Young: 9 Old: 32	Feeding behavior monitoring	· The old group exhibited significant alterations in feeding behavior compared with the young adult group, characterized by a greater number of bites, shorter inter-bite intervals, and a longer time required to consume pasta, indicating age-related impairment in mastication efficiency. · Aging was associated with increased variability in feeding duration, but not in bite number or inter-bite interval variability.
Kletzien et al. (2019) [41]	Fischer 344/Brown Norway rats (-) ^a^	Young: 6 Old: 29	VFSS/swallowing function	· With aging, the bolus area significantly increased, whereas the mastication rate was significantly decreased in the old group than in the young adult group. However, no significant difference existed in bolus velocity between the two age groups. · Aging was associated with reduced tongue base retraction, more variable masticatory movements, and compensatory head movements (e.g., increased flexion and caudal movement of the head) in old rats, compared with young rats, during swallowing.
Randolph et al. (2014) [42]	FVB mice (male and female)	Young: 6 Old: 24	Lick assay	· From 6 months to 24 months of age, lick rates significantly decreased.
Lever et al. (2015) [43]	C57BL/6 mice (female and male)	Young: 4–7 Old: 18–21	VFSS	· Among 15 swallow metrics, six metrics showed significant age-related differences. The old group had significantly longer pharyngeal and esophageal transit times, required larger bolus volumes to trigger the pharyngeal swallow response, and had a higher percentage of ineffective primary esophageal swallows. Additionally, the old group had a significantly slower lick rate, measured via tongue and jaw cycle rates. · The old group exhibited a generally slower swallowing process across all stages: oral, pharyngeal, and esophageal.
Mogami et al. (2017) [44]	C57BL/6 mice (male)	Young: 7 weeks Old: 23–25	Feeding behavior monitoring	· Total meal amounts and meal frequencies did not differ between the young and old groups. However, total meal time was significantly longer in the old group. · The old group had a significantly higher bout frequency and average bout frequency than the young group. By contrast, the average bout size and average bout time per bout were significantly smaller, whereas the average interbout interval was significantly increased in the old group.
Takahashi et al. (2025) [45]	C57BL/6 mice (male)	Young: 16 weeks Old: 77 and 104 weeks	VFSS	· With aging, the bolus area significantly increased, while the lick rate progressively decreased into old age. · No significant age-related differences were observed in the lick–swallow ratio, interswallow interval, or pharyngeal transit time.

VFSS: videofluoroscopic swallowing study. ^a^ The minus symbol (-) indicates “not mentioned”.

## Data Availability

No new data were created or analyzed in this study. Data sharing is not applicable.

## Abbreviations

The following abbreviations are used in this manuscript:

PRISMA	Preferred Reporting Items for Systematic Reviews and Meta-Analyses
SYRCLE	Systematic Review Centre for Laboratory Animal Experimentation
GG	Genioglossus
HG	Hyoglossus
SG	Styloglossus
MHC	Myosin heavy chain
CSA	Cross-sectional area
NMJ	Neuromuscular junction
SCs	Satellite cells
5HT	Serotonin (5-hydroxytryptamine)
ET	Extrinsic tongue muscle
IT	Intrinsic tongue muscle
IHC	Immunohistochemistry
SDS-PAGE	Sodium dodecyl sulfate polyacrylamide gel electrophoresis
WB	Western blotting
RT-qPCR	Reverse transcription quantitative polymerase chain reaction
MyoD	Myoblast determination protein
BDNF	Brain-derived neurotrophic factor
TrkB	Tropomyosin receptor kinase B
TA	Thyroarytenoid
PCA	Posterior cricoarytenoid
LCA	Lateral cricoarytenoid
CT	Cricothyroid
VFSS	Videofluoroscopic swallow studies
H&E	Hematoxylin and Eosin
